# Phenotyping Tomato Root Developmental Plasticity in Response to Salinity in Soil Rhizotrons

**DOI:** 10.34133/2021/2760532

**Published:** 2021-01-20

**Authors:** Jacinto Gandullo, Safarina Ahmad, Essam Darwish, Rumyana Karlova, Christa Testerink

**Affiliations:** ^1^Section of Plant Physiology and Plant Cell Biology, Swammerdam Institute for Life Science, University of Amsterdam, Science Park 904, 1098 XH Amsterdam, Netherlands; ^2^Departamento de Biología Vegetal y Ecología, Área de Fisiología Vegetal, Facultad de Biología, Universidad de Sevilla, Seville, Spain; ^3^Plant Physiology Section, Agricultural Botany Department, Faculty of Agriculture, Cairo University, 12613 Giza, Egypt; ^4^Laboratory of Plant Physiology, Plant Sciences Group, Wageningen University and Research, 6708PB Wageningen, Netherlands

## Abstract

Plants have developed multiple strategies to respond to salt stress. In order to identify new traits related to salt tolerance, with potential breeding application, the research focus has recently been shifted to include root system architecture (RSA) and root plasticity. Using a simple but effective root phenotyping system containing soil (rhizotrons), RSA of several tomato cultivars and their response to salinity was investigated. We observed a high level of root plasticity of tomato seedlings under salt stress. The general root architecture was substantially modified in response to salt, especially with respect to position of the lateral roots in the soil. At the soil surface, where salt accumulates, lateral root emergence was most strongly inhibited. Within the set of tomato cultivars, H1015 was the most tolerant to salinity in both developmental stages studied. A significant correlation between several root traits and aboveground growth parameters was observed, highlighting a possible role for regulation of both ion content and root architecture in salt stress resilience.

## 1. Introduction

Soil salinization is a growing problem for agriculture worldwide. More than 6% of the world's total land area is salinized, and this percentage is higher (about 25%) in arid or semiarid zones of the Earth [1, 2]. The salinization of land is due to natural and anthropic causes including climate change-related higher evaporation, watering with saline water, and poor agricultural practices [1]. High salinity of the soil is considered one of the most severe environmental stresses which cause crop yield loss and low food quality products [3]. Plants have developed a wide range of strategies to sense and respond to salt stress. These responses involve different mechanisms that mediate the ability of the plant to withstand the deleterious effects of salinity, yet they often have consequences for plant productivity [1, 3].

Plant roots are the first organs that detect salinity [4]. When the salt concentration increases around the root zone, the plant response can be subdivided in two phases: early-occurring osmotic stress which reduces the availability of water uptake and a slower response due to the accumulation of ions in plant tissues (ionic stress) which affects nutrient uptake and balance and ion homeostasis [1, 3, 5]. Root system architecture (RSA) plasticity is a consequence of the integration of environmental cues into the root developmental program [6, 7]. Stress signals modulate RSA through changes in primary root growth and lateral root (LR) development, although LR formation is considered the major determinant controlling the RSA [8, 9]. Different root architecture traits are currently being used in breeding programs for improvement of yield and stress tolerance in crops [10, 11].

In Arabidopsis, natural variation exists between root architectural responses of different accessions analyzed [12]. Salt stress was shown to influence the rates of lateral root emergence, and the main root vector angle and straightness [13]. The level of salinity is another important factor in the root response; high salt stress (≥100 mM) induced an arrest of the main root (MR) and LR growth; however, mild salinity could even stimulate root growth [11, 12, 14]. Another clear example of root plasticity is the halotropic MR growth response to avoid salt [8]. Although the importance of plant roots in sensing and responding to salt stress is recognized and these results suggest that modulation of root architecture can be used as a strategy to achieve salt stress resilience, what is lacking currently is knowledge of RSA plasticity of crop plants.

For tomato (*Solanum lycopersicum*), one of the most important vegetable crops worldwide, production is concentrated in warm and semiarid areas where the climate conditions are optimal, but frequently, the soils are affected by salinity. In these areas, the selection of optimal tomato cultivars with enhanced salt tolerance is essential to maintain yield and productivity [15–17]. Domesticated tomatoes are classified as glycophytes, considered moderately salt sensitive when compared to their more resilient wild relatives. In different wild tomato species (*S. peruvianum* and *S. pennellii*), salt tolerance is in part associated with a reduction of root biomass [18]. A recent genome-wide association study identified a significant association between the gene *SlHAK20*, which encodes a Na^+^ and K^+^ transporter, and root Na^+^/K^+^ ratio. The authors suggest that natural variation in *SlHAK20* could be responsible for the loss of salt tolerance during tomato domestication [19]. Even within the domesticated tomatoes, the variability in salinity sensitivity is relatively high [19–21]. Several other tomato genes involved in salt resilience have been identified, including ion transporters *SlHKTL2* and *SlNHX3* [2, 22, 23] and a signaling protein SlCBL10 [24]. The relative importance of the different processes involved in salt tolerance is dependent on the genotype, time of exposure, environmental condition, and salt concentrations used [1, 5].

Although grafting experiments have shown that the rootstock contributes to fruit yield and quality under salt stress [25], for tomato, we have very little knowledge about gene regulatory networks and physiological processes controlling the formation and plasticity of RSA under salt stress. In a study on tomato seedlings grown on plates containing standard Musharige and Skoog medium with agar (MS agar) and treated with salt, the main root length (MRL) was inhibited only at high salinity in the most salt-sensitive genotype tested, but not in the salt-tolerant genotype LA2711, showing a clear genotypic variation for this trait [26]. These data suggest that MRL might serve as a good indicator of tomato salt tolerance [26].

Different methodologies have been used to study RSA in response to salt stress [27]. Most of the studies on RSA in salinity stress conditions employ soil-free techniques such as agar plates, hydroponics, or paper pouches [12, 13, 28, 29]. These methods allow for better control of the experimental variables but are considerably far from the field conditions [27]. Soil is a very heterogeneous nonsterile environment and has a strong influence on the root growth more similar to field conditions, but with complicated access to monitor the intact root system for analysis [6, 27]. Several research groups have developed sophisticated platforms for root phenotyping using luminescence or transparent soil-filled chambers or automated phenotyping methods where rhizotrons are placed at an angle and a low compacted soil is used as a substrate [30–32].

Here, in order to characterize the RSA of tomato plants in response to controlled salinity, a cheap and simple nonautomated method of root phenotyping, using soil as a substrate, was developed. We present a phenotyping method which allows studying plasticity of an intact root system in soil, but does not require expensive equipment. Using this soil rhizotron method, further referred to as rhizotron in this manuscript, we analyzed and compared RSA of five *Solanum lycopersicum* commercial genotypes under both nonsaline and saline conditions, revealing substantial plasticity of the tomato root system. To test the putative relationship between RSA and salt tolerance response, several parameters related to salt tolerance were evaluated, which revealed root ion content as a novel parameter of interest. In addition, salt tolerance was analyzed at different developmental stages in order to identify possible stage-specific salt tolerance responses in the selected tomato varieties.

## 2. Materials and Methods

### 2.1. Plant Materials and Growth Conditions

Seven *Solanum lycopersicum* genotypes were used in this study. Walter, Moneymaker, and LA0147 are old indeterminate cultivars. H8504, H9661, H1015, and H5003 are four determinate hybrid varieties developed by Heinz and were kindly provided by Conesa (Spain). Heinz lines are currently used by the food industry in Spain for processed tomato products.

Tomato plants were grown with a 16/8 photoperiod at 24°C and 60% of humidity. The experiments were set up in a randomized design with 10-20 replicates per treatment and per genotype.

The seeds were surface sterilized in two steps with 4% of commercial bleach and 20 mM of HCl and washed several times with sterilized water after every step. For rhizotron and pouches, sterilized seeds were germinated on Petri dishes for 3-4 days on a moist sterile filter paper at 24°C in darkness. After germination, seedlings with a radicle approximate of 1 cm were transferred to different phenotyping systems. For the pouches, 4-day-old seedlings were transferred to large paper pouches purchased from Phytotc (CYG-98LB) and treated with a quarter strength liquid Musharige and Skoog medium (MS) [33] supplemented or not with 120 mM NaCl. For rhizotron system, transparent square plates (245 × 245 × 25 mm, Thermo Fisher Ref. 240835) were filled with a mixture of soil and sand. The soil was a standard substrate made of Swedish peat moss and supplied by Jongkind. The plates were wrapped around with aluminum foil and placed vertically at a 70° angle. The plants were irrigated with 100 ml of water (control) or 100 ml of 120 mM NaCl solution. Detailed information about the different rhizotron setup can be found in Materials and Methods S1.

For the agar plates, sterilized seeds were germinated and grown in control plates containing a quarter strength MS medium including vitamins with 1% of Daishin agar (Duchefa). After 4 days, seedlings were transferred to big square plates (245 × 245 × 25 mm) with MS medium including vitamins supplemented or not with 120 mM of NaCl.

For experiments with older tomato plants, seeds were germinated on soil in a small box. After one week, seedlings were transferred into 5 liter pots and grown for 3 more weeks. One-month-old plants were irrigated with 300-500 ml of water (control) or a salt solution (salt) three times per week during 4 weeks. The salt concentration in the saline solution was increased with 30 mM per treatment until it reached 120 mM of salt. 30-day-old plants were treated for 30 more days and then were harvested. From each plant, the 4^th^ and the 10^th^ vital leaves from the bottom were harvested (old and young leaf samples, respectively) for proline and relative water content (RWC) determination.

### 2.2. Phenotyping

**Figure 1:**
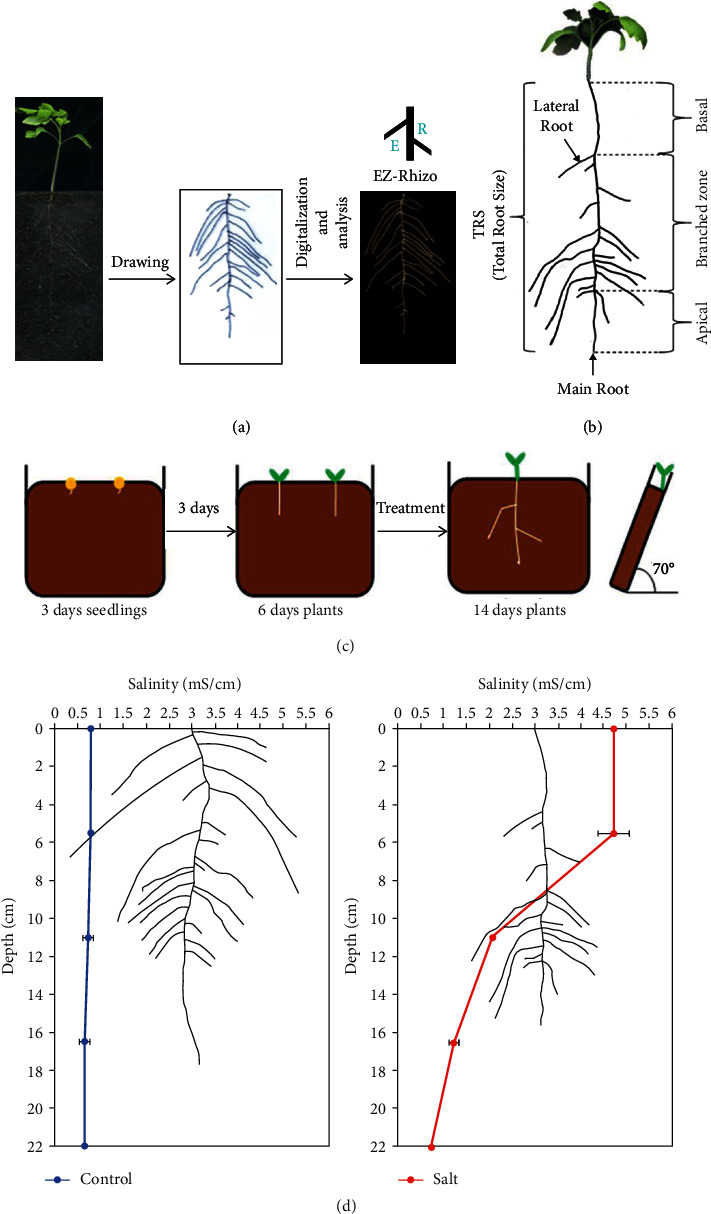
Tomato root traits and analysis. (a) Acquisition, processing, and analysis of roots from rhizotron. EZ-Rhizo software was used to measure the root traits. (b) Schematic representation of tomato root traits. Total root size is the sum of the main root length and lateral root length. (c) Illustration of the growth system rhizotron and the final setup used for Figures 3–6. Three-day-old seedlings were transferred to soil plates, and 3 days after the transfer, the seedlings were treated with 120 mM NaCl (salt) or tap water (control). The treatment was repeated every 4 days. Finally, the plants were analyzed and harvested for 14-day-old seedlings. (d) Superposition of root system architecture and soil salinity distribution along the rhizotron under control and salt conditions. Salinity values represent the mean ± SE of 6 replicates of two independent experiments.

For RSA analysis, roots from 10- or 14-day-old plants were drawn on transparent plastic sheets and root images were scanned at a resolution of 200 dpi using an Epson scan (Epson perfection V550 photo). The images were first opened with the ImageJ software to invert the color. Finally, root images were analyzed using EZ-Rhizo software (Figure 1(a)) [34]. The root traits analyzed were the main root length (MRL), number of lateral root (NLR), total root size (TRS), lateral root size (LRS), lateral root density of the main root (LRD-MR), basal zone length (Basal); branched zone length (Branched), and apical zone length (Apical) (Figure 1(b)).

### 2.3. Soil Sampling and Analysis

The soil plates were divided into 4 sections of 5 cm of depth, and 4 random samples of every section were collected. Soil samples were dried in an oven at 70°C for 2 weeks and lightly ground. Soil electrical conductivity (EC) (1 : 5) was determined according to [35]. Salinity measurements were done using a conductivity meter Cond3110 (WTW, Xylem Analytics).

### 2.4. Plant Biomass Measurement

For biomass measurements, leaves and stems were processed separately. Each tissue was weighed after harvesting to obtain the fresh weight (FW). After that, plant tissues were dried in an oven for 1 week at 65°C and weighed again to quantify the dry weight (DW). The relative water content (RWC) was also calculated with the formula RWC = ((FW − DW)/FW)∗100.

### 2.5. Proline Measurement

For proline quantification, 50 and 100 mg of fresh material from the control and salt-treated plants were used. Extraction and quantification were done as described before [36].

### 2.6. Ion Content Measurement

Na^+^ and K^+^ ion content was measured in the leaves, stems, and roots using flame photometry. Fresh tissue was rinsed and dried in an oven at 70°C for 48 h. Finally, dry tissue was digested and ion content was quantified as described by Plett et al. (2010) [37].

### 2.7. Statistics

All data were analyzed by ANOVA, and means were compared by Duncan's multiple range test. A *p* value of < 0.05 was considered to be statistically significant. Pearson correlation coefficients squared were calculated on average values. The size of the correlation coefficient was interpreted according to [38]. All analyses were conducted using SPSS Statistics 25 (IBM).

## 3. Results

### 3.1. RSA Phenotyping in Rhizotrons, Paper Pouches, and Agar Plates

**Figure 2:**
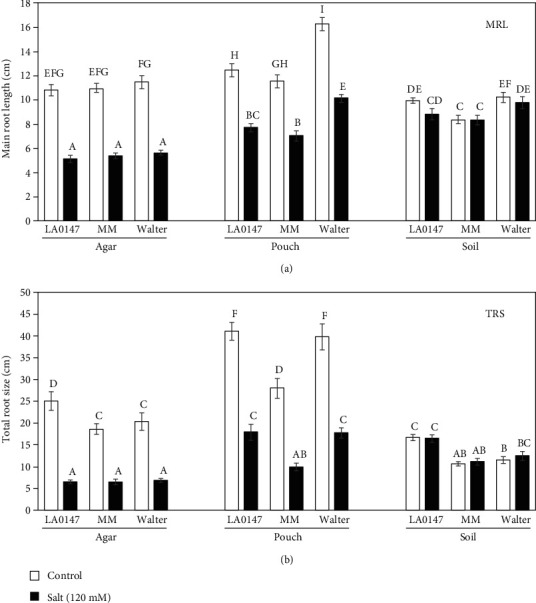
Comparison of root phenotyping methods in tomato. Root system architecture traits were analyzed in three tomato cultivars under salt or control conditions in agar plates, pouches, or rhizotrons: (a) MRL: main root length; (b) TRS: total root size. Roots of 10-day-old plants treated or not with salt for 6 days were analyzed with EZ-Rhizo software. The data represent the mean ± SE (standard error) of 20 replicates from two independent experiments. Different letters within each panel indicate significant differences according to Duncan's multiple range test, *p* < 0.05.

In order to optimize the rhizotron method for RSA phenotyping, 4-day-old seedlings were treated once with or without salt solution and grown for 6 more days. We compared the rhizotrons with paper pouches and agar plates (Figure S1). Our data showed that the root development was faster on the pouches compared to the soil and plates in control conditions for all genotypes. Under salt conditions, the reduction of the main root growth and other RSA traits was bigger on the agar plates in comparison to the pouches or rhizotron. The rhizotrons showed reduced root growth compared to the other methods but interestingly were the least affected by salinity (Figure 2 and S2). Thus, under this experimental setting, the deleterious effect of salt treatment on root growth in rhizotrons was less severe compared to the agar plates or paper pouches.

We next tested the rhizotron system, starting with transferring 3-day-old seedlings and a prolonged treatment of 6-day-old seedlings. With this setup, we could phenotype tomato plants grown for two weeks, treated twice for in total of 8 days with 120 mM NaCl (Figure 1). Measuring the soil salinity along the rhizotron showed that under nonsaline conditions (control), the salt level was low and uniform, while under saline conditions, a gradient was observed. The salinity distribution in the soil was high at the surface of the rhizotron and lower at the bottom (Figure 1(d)).

### 3.2. Root Phenotyping in Rhizotrons Shows RSA Plasticity of Different Tomato Varieties under Salt Stress

**Figure 3:**
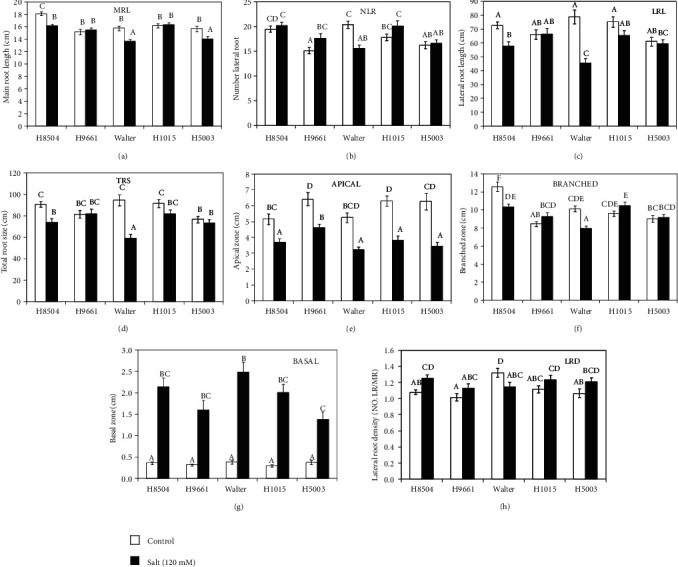
Root system architecture trait analysis of five tomato cultivars under salt stress (salt) or nonstressed conditions (control). Plants were grown on rhizotrons as is indicated in Figure 1(a) and treated or not with 120 mM of NaCl at 6 days and 10 days after germination. Root system was analyzed of 14-day-old plants. (a) MRL: main root length; (b) NLR: number of lateral roots; (c) LRL: lateral root length; (d) TRS: total root size; (e) Apical: apical zone length; (f) Branched: branched zone length; (g) Basal: basal zone length; (h) LRD: lateral root density per main root. Data represent the mean ± SE of 40 replicates of two independent experiments. Different letters within each panel indicate significant differences according to Duncan's multiple range test, *p* < 0.05.

A comparative study on the effect of salt stress on seven root architecture traits was carried out in five tomato genotypes, four hybrid tomato cultivars H8504, H9661, H1015, and H5003, and the commonly used genotype Walter (Figure 3). In general, in all genotypes, the salinity led to a significant reduction of the apical zone size and a strong increase of the basal zone size of the main root (Figures 1(d), 3(e), and 3(g)). Under salt stress, apical zone inhibition did not show any clear genotypic differences, while the root basal zone induction by salt was the highest in Walter and the lowest in H5003. The main root length (MRL) was significantly reduced in H8504, Walter, and H5003 under salinity (Figure 3(a)). H8504 and Walter cultivars also exhibited a reduction in lateral root length (LRL) and total root size (TRS) under saline conditions (Figures 3(c) and 3(d)). The differences for LRL and TRS growth reduction under salt stress were mostly visible for Walter. Salt treatment caused a lower number of lateral root (NLR) in Walter, but in H9961, for example, the effect was opposite; the NLR was increased in salt. Only H8504 and Walter showed a decrease in the branched zone size when the plants were subjected to salinity. Lateral root density (LRD) was calculated relative to MRL. Under salinity, LRD decreased in Walter due to the lower NLR, but in H8504, LRD was higher than the control, due to the more severe reduction in MRL in this condition (Figures 3(a) and 3(h)). In summary, our root phenotyping method allows a noninvasive analysis of 2D RSA in tomato on soil that reveals significant differences in RSA modulation between genotypes tested.

### 3.3. Salinity Resilience Differs between the Cultivated Tomato Varieties Tested

**Figure 4:**
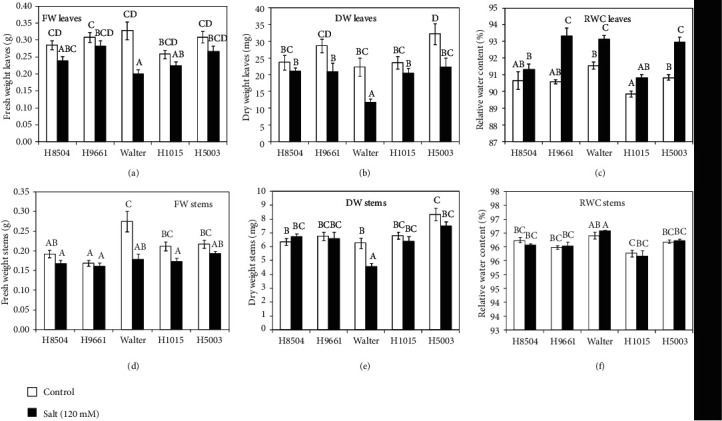
Growth parameters. Characterization of growth parameters of tomato cultivars under salt (salt) stress or nonstressed conditions (control) in leaves (upper row) and stems (bottom row). (a, d) FW: fresh weight; (b, e) DW: dry weight; (c, f) RWC: relative water content. Plants were harvested 14 days after germination and treated or not with 120 mM NaCl for 8 days. Each value represents the mean ± SE of 30 replicates from 2 independent experiments. Values marked with different letters within each panel are significantly different according to Duncan's multiple range test, *p* < 0.05.

Plant growth and biomass yield are classically used to evaluate plant tolerance to salt stress [39]. Therefore, we assessed these parameters in the rhizotron-grown plants. Salt stress treatment significantly reduced the above ground dry weight (DW) for Walter, H9661, and H5003 cultivars (Figure 4(b)). For H9661 and H5003, this reduction in leaf DW was linked to a higher relative water content (RWC), so no differences were observed in leaf fresh weight (FW) (Figures 4(a)–4(c)). Leaf FW was only reduced by salinity in Walter. In contrast, no differences in leaf FW were found for H8504 and H1015 under either control or salt condition. For the stems, only Walter showed a clear decrease in biomass, affecting not only the DW but also FW in the saline conditions, while no differences in RWC of the stem were observed between the control and salt conditions (Figures 4(d)–4(f)).

**Figure 5:**
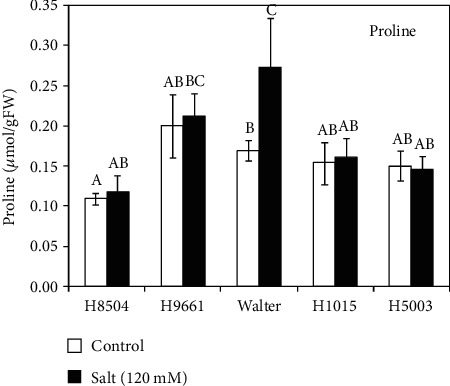
Effect of salt stress on proline concentration in leaves of five tomato cultivars. Leaves were collected from 14-day-old plants treated or not with 120 mM NaCl for 8 days. Each value represents the mean ± SE of 8 replicates from 2 independent experiments. Different letters indicate statistically significant differences according to Duncan's multiple range test, *p* < 0.05.

**Figure 6:**
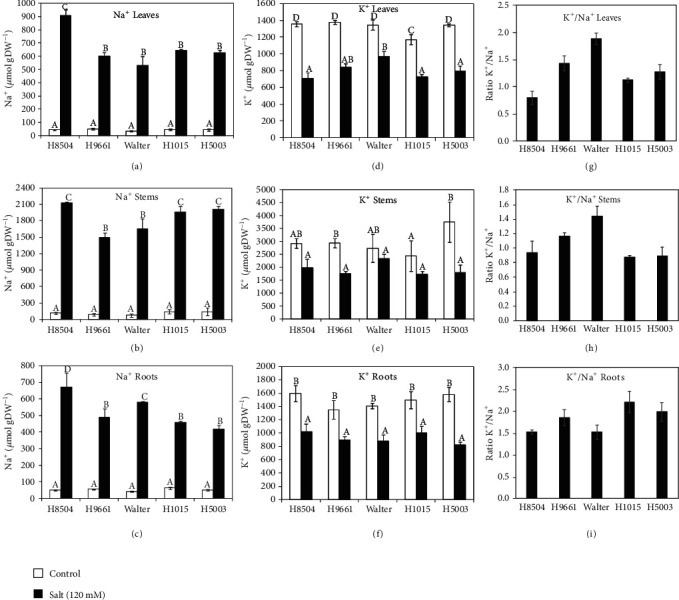
Effect of NaCl on Na^+^ and K^+^ concentration in tomato. (a–c) Na^+^ concentration (*μ*mol·gDW^−1^), (d–f) K^+^ concentration (*μ*mol·gDW^−1^), and (g–i) K^+^/Na^+^ ratio in leaves (upper row), stems (medium row), and roots (bottom row) of 14-day-old tomato plants treated with 0 and 120 mM NaCl for 8 days. Values represent the mean ± SE of 6 replicates from 2 independent experiments. Different letters within each panel indicate significant differences according to Duncan's multiple range test, *p* < 0.05.

Salinity stress causes the accumulation of the amino acid proline serving as an osmoprotectant in several species including tomato [40]. Therefore, we measured proline concentration in leaves of control and salt-treated plants (Figure 5). After eight days of treatment with 120 mM salt, only Walter showed a slight increase in proline concentration in salt-treated plants. Our data thus suggests that the differences in salt resilience observed between the different tomato varieties are likely not due to proline accumulation. We next determined the ion content in leaves, stems, and roots for the five cultivars tested under control and salt conditions (Figure 6). In all plant parts from the five genotypes, Na^+^ content was significantly increased after eight days of salt treatment compared to the nonsaline conditions. In general, under salt stress, the highest Na^+^ content was measured in stems, while leaves and roots showed similar levels of Na^+^. Interestingly, the greatest increase in Na^+^ ions under salinity was observed in the leaves and roots of H8504, which did not show a significant reduction in shoot growth, but several RSA parameters were affected under salt stress compared to the control conditions in this cultivar (Figures 3 and 4). H9661 and Walter had the lowest Na^+^ content in the stems under salinity. In general, K^+^ content decreased under salinity stress; however, in the stems of H8504, Walter, and H1015 varieties, no significant differences were observed in the K^+^ concentration between the control and salt-treated plants. Under salinity, genotype differences were only found in leaves, where Walter accumulated higher K^+^ than other genotypes. We further calculated the K^+^/Na^+^ ratio in different tissues. After eight days of salt treatment, all genotypes showed low values for K^+^/Na^+^ ratio in comparison with control samples, as expected. However, in the leaves and stems, Walter presented the highest K^+^/Na^+^ ratio under salt conditions. Although increased K^+^/Na^+^ ratio is considered to contribute to salinity resilience, in the case of Walter, this observation did not correlate with the observed high salt sensitivity regarding RSA traits and shoot growth (Figures 3 and 4).

### 3.4. Correlation Analysis between RSA Traits and Other Parameters Studied

**Figure 7:**
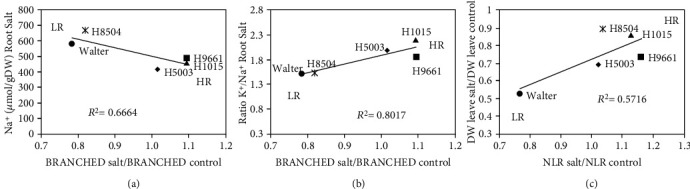
Relationship between salt tolerance parameters and RSA traits. (a) Na^+^ content in root salt and branched zone. (b) Ratio K^+^/Na^+^ in root salt and branched zone. (c) DW: dry weight of leaves and NLR. DW leaves and RSA traits were represented as the ratio between salt divided by control values. Different symbols represent the five genotypes. Correlation between pairs of variables was tested using the Pearson correlation coefficient squared (*R*^2^). Statistically significant correlations are shown by an asterisk ∗, *p* < 0.05. HR: high resiliency area; LR: low resiliency area.

To evaluate a potential relationship between RSA reduction and ion content, a correlation analysis was performed. Interestingly, these results showed a significant correlation between branched zone reduction and the root ion traits Na^+^ accumulation and K^+^/Na^+^ ratio (Figures 7(a) and 7(b)). Moderate correlations were also found between root ion parameters and most other RSA traits (Figure S3). For shoot reduction in response to salt stress, only NLR showed a significant correlation (Figure 7(c)).

### 3.5. Tolerance Response in Long-Term Salt Treatment

**Figure 8:**
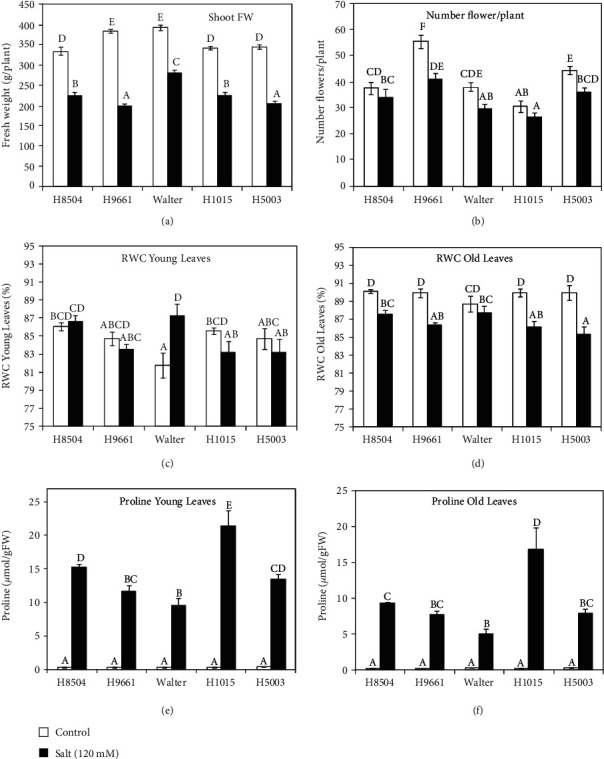
Effect of the salinity on salt tolerance in long-term treatment of five tomato cultivars. Plants were grown in soil in pots for 30 days after germination, subsequently treated for 30 days with water (control) or 120 mM NaCl (salt) and harvested at 60 days after germination. (a) Biomass yield as shoot fresh weight (FW); (b) number of flowers per plant; (c, d) RWC: relative water content; (e, f) proline accumulation. (c, e) Young leaves and (d, f) old leaves (10^th^ and 4^th^ leaves from the bottom, respectively) were analyzed separately for RWC and proline content. Each value represents the mean ± SE of 10 replicates. Values marked with different letters within each panel are significantly different according to Duncan's multiple range test, *p* < 0.05.

To analyze the effect of the developmental stage on the salt tolerance in the different tomato cultivars, several classic physiological parameters related to salt tolerance were measured. The plants, 60 days old, grown in pots were treated with salt for 30 days. Our data showed that the salt stress treatment significantly decreases the shoot growth of each genotype (Figure 8(a)). The genotypes that showed higher sensitivity in response to salinity were H9661 and H5003, exhibiting, respectively, 48 and 41% of shoot reduction (Figure S4(a)). The number of flowers per plant was used as a proxy for evaluating the plant yield (Figure 8(b)). For this trait, genotypic differences were found as well, where H9661, Walter, and H5003 were the cultivars that showed a significant decrease in the number of flowers compared to the control under salt condition (Figure S4(b)). No significant differences were observed in the number of flowers for H8504 and H1015. Concerning the RWC, only Walter showed a difference in response to salt stress in young leaves (Figure 8(c)). In this genotype, the salinity stress increased the relative water content in young leaves RWC. It suggests that Walter could have a better control of the water relation parameters and is able to maintain a good water content under salt stress [39]. Nevertheless, in old leaves, the salinity led to reduced RWC in all genotypes except in Walter which did not show any difference in RWC compared to the control (Figure 8(d)). In the long-term saline treatment, the proline content was significantly increased by salinity in both young and old leaves from every genotype (Figures 8(e) and 8(f)). The levels of proline were lower in the old leaves for all genotypes compared to the young leaves. H1015 variety showed the highest proline accumulation in leaves, while Walter had the lowest.

The data presented here showed that from all cultivars tested, H1015 is the most resilient to salt stress in both short and long stress treatments, and exhibits high K^+^/Na^+^ ratio in the roots and high proline accumulation.

## 4. Discussion

Soil salinity is an important and increasing problem for modern agriculture. Breeding programs in crops are often focused on shoot-related traits; however, salinity directly influences the root system [11, 41]. Multiple studies, using model plants, have described the genetic mechanisms that underlie RSA, but less is known about optimal root phenotypes for crops. One of the main factors in RSA studies is to select an optimal RSA phenotyping method. Artificial conditions (agar or hydroponic) are used as easy and noninvasive root phenotyping platforms for seedlings; however, the results can be difficult to be extrapolated to soil conditions.

A rhizotron setup represents a more natural environment for plant roots and involves the mechanical impedance which is related to the soil properties and that has an important effect on root development [32]. This mechanical impedance could explain the slower root growth rates in rhizotron in comparison with agar plates or pouches. In this paper, we describe a low-cost rhizotron platform for a 2D, noninvasive method of RSA analysis for tomato seedlings. This phenotyping method uses plastic plates with a thin layer of soil as substrate.

In the present work, RSA parameters related to root growth (MRL, LRL, and TRS) were analyzed, showing a clear reduction in the cultivars Walter and H8504 in response to salinity and a significant genotypic variation for these traits. In *Arabidopsis*, the effect of salt on NLR is highly variable depending on whether the dynamics of LR development is considered in the analysis. Yet in general, salt arrests LR emergence [11, 12, 14, 42]. For tomato, after 8 days of salt treatment, we found that only Walter showed a significant reduction in the NLRs, while in the H9661 variety, we observed an increase in saline conditions. Further, NLR could be investigated as a useful parameter to predict salt tolerance because of the fact that a significant correlation with shoot growth reduction was observed (Figure 7).

In Arabidopsis, it was shown that the pattering of LRs is determined in the root tip, and LR emergence in the region of MR grown before the transfer to salt condition was not affected [12, 42, 43]. On the other hand, after the seedlings were transferred to saline medium, LR emergence was strongly inhibited and the number of LR decreased. Here, the most prominent response of tomato RSA in rhizotrons was a change in pattering of LRs on the MR in response to salt. Our data showed that the main suppression of LR emergence by salt in tomato was at the basal zone, the MR region that developed before the salt treatment, and as a consequence a larger basal zone size was observed. These data suggest that in tomato, the mechanism through which stress affects the patterning of LRs may be regulated in a different manner compared to *Arabidopsis.* Together, our results reveal high plasticity of tomato roots under salinity; RSA was largely remodeled compared to nonsaline conditions.

Interestingly, we observed that in the rhizotron plates treated with saline water, the salt accumulates at the soil surface producing a strong salinity gradient in the soil. Similar results were found in field conditions when several crops were irrigated with saline water by drip or sprinkler irrigation [44, 45]. At the soil surface, where the highest salinity concentration was measured, lateral root emergence was most strongly inhibited resulting in an increased basal zone size. As this response was also found in agar plates and pouches (Figure S2(d)) that present a more uniform salt concentration, this could be an adaptive response to the natural conditions in saline soils, rather than the consequence of local differences in salt concentration in the setup. For a root trait to be used in breeding programs for salt tolerance, the existence of genetic variability in a bank of germplasm is a prerequisite [21, 46]. Among all root traits analyzed here, MRL, LRL, TRL, NLR, basal, and branched zone size showed genotypic variations in response to salinity and might be potential candidates for evaluation of tomato salt tolerance. We show that NLR and especially branched zone present a significant correlation with different parameters related with salt tolerance (Figure 7).

Salt tolerance is a complex trait that involves different aspects of the genetic architecture, biochemistry, and physiology of the plant. Soil salinity is known also to reduce shoot growth in response to salt stress because salinity affects the water homeostasis and ion distribution [1, 47]. Previous work in tomato plants and seedlings has reported that salinity has a negative impact on shoot growth [48–50]. Under our experimental conditions, not all genotypes were affected in shoot biomass by salinity and a wide genotypic variation was found. Walter was the most sensitive cultivar showing a significant reduction of all growth parameters in both leaves and stems, while in H8504 and H1015, no differences were observed in shoot biomass in response to salt stress. It is well established that compatible organic solutes such as proline are accumulating in response to salt stress in plants including tomato [1, 39, 47, 51]. However, here, we observed only a slight increase in proline accumulation in the short-term salt treatment. Eight days of salt treatment under our experimental conditions appears insufficient for a clear increase in proline synthesis and accumulation.

In general, salinity produces an ion imbalance as a result of an excessive Na^+^ uptake and a reduction in K^+^ concentration [21, 40, 48, 52]. Salt tolerance is associated with the capacity of the plant cells to maintain ion homeostasis under salt stress [47]. Plants have developed different strategies or adaptations to avoid ion toxicity: excluder plants exclude Na^+^ accumulation in the shoots, while includer plants accumulate high Na^+^ concentration in shoots [16, 21, 52]. Our data show that all cultivars present a similar Na^+^ distribution in the different tissues analyzed. Na^+^ was mainly accumulated in stems, suggesting that all genotypes studied here present an includer salt tolerance strategy, under our experimental conditions [16, 21]. A negative correlation between salt tolerance and Na^+^ content in the leaves has been described in different species including tomato [53, 54]. Nevertheless, our results did not show a positive relation between leaf Na^+^ accumulation and salt tolerance which is in agreement with other previous reports in *Arabidopsis* and tomato [55, 56]. Also, for shoot K^+^/Na^+^ ratio, we did not observe a positive relation with biomass production or salt resistance.

While no clear relation between root ion accumulation and salt tolerance was found previously in tomato in hydroponics setups [21, 40], we here show intriguing results that highlight a possible role for ion homeostasis in the root, rather than the shoot, for salt tolerance. The most salt-sensitive cultivars with respect to root growth, Walter and H8504, showed high Na^+^ content in the roots and the lowest root K^+^/Na^+^ ratio, while the most salt-tolerant cultivar H1015 had the highest K^+^/Na^+^ ratio in the root. In addition, both root ion parameters were significantly correlated with branched zone size reduction. Recently, it was observed that tomato plants with a broad root system also had a reduction in soil salinity at the root zone in comparison with confined root systems, presenting a more favorable condition for plant development [57]. In accordance with these results, we found a negative correlation between root Na^+^ content and root size. Summarizing, MRL, LRL, and LR distributions (branched zone) seem to be the most promising RSA parameters in order to quantify root volume and size, and to evaluate salt tolerance in tomato. Other studies have also highlighted the importance of RSA in salt tolerance. In *Arabidopsis* and rice, a relation was suggested between LR traits and ion content parameters in shoot; however, a potential relation with root ion content was not analyzed [12, 58]. Also in tomato, most of the salt tolerance indicators are related to the shoot (aboveground) part of the plant [46]. Our results highlight the importance of not only root system architecture but also root ion content, in tomato response to salt stress, which provides an interesting new avenue to explore further for salinity tolerance mechanisms and breeding in tomato.

It has been shown that salt tolerance generally changes with plant age in tomato and other crops, indicating that salt tolerance is developmentally regulated and has a stage-specific response [17, 59]. According to previous results, long treatment of tomato cultivars induced a general decrease in shoot biomass, although genotypic differences were found [21, 49]. Our data showed that the most sensitive genotypes to long salinity treatment were H9661 and 5003 because they showed high reductions in both yield traits analyzed. On the other hand, the most tolerant cultivars were 8504 and H1015. Walter showed a high reduction in the number of flowers but less in shoot fresh weight suggesting a moderate salt tolerance. Tolerance assays with older plants confirmed that salt tolerance has a developmental-stage dependence, and different salt resilience was observed in several genotypes depending on plant ageing. Despite this dependence, H1015 and Walter showed similar response to salt stress when salt tolerance parameters, from long- and short-term assays, were analyzed together. H1015 was the most salt-tolerant genotype, while Walter presented high sensitivity to salinity in both developmental stages.

In summary, we show that rhizotrons provide an efficient and affordable root phenotyping platform for tomato seedlings. It simulates, at a small scale, the natural environment of the plant, forming a salinity gradient similar to saline soils, and the method could be scaled up in automated phenotyping setups. Using the rhizotrons, we reveal a high level of plasticity in the response of roots to salinity, leading to remodeling of root architecture. Lateral root emergence was inhibited in the root basal zone, which is placed in the upper part of the soil corresponding to the highest salt concentration both in salt-treated rhizotrons and natural field conditions. Several correlations were observed among the different root traits, including ion content and salt tolerance. Therefore, RSA parameters as well as ion content in roots might be considered as good candidate traits to analyze for future application in breeding programs for salt resilience.

## Data Availability

The original image data used to support the findings of this study are available from the corresponding author upon request. All other data used to support the findings of this study are included within the article or within the supplementary information files.

## Supplementary Materials

Supplementary Materials Supplementary Materials and Methods S1. For rhizotron phenotyping, two different protocols were carried out. In the preliminary setup (result from Figure 2 and S2), 4-day-old seedlings were transferred to soil plates and irrigated once with 100 ml of water (control) or 100 ml of 120 mM NaCl (salt) solution. RSA was analyzed for roots of 10-day-old plants that were treated for 6 days. In the final setup designed for rhizotron phenotyping and represented graphically in Figure 1(a), 3-day-old seedlings were transferred to soil plates, and 3 days after the transfer to soil, seedlings were irrigated with 100 ml of tap water (control) or 100 ml of 120 mM NaCl (salt) solution per plate. The treatment was repeated every 4 days from the first treatment in the same conditions. In this protocol, two treatments were done, at 6 days and 10 days after germination. Finally, the plants were harvested and analyzed at 14 days old stage and treated for 8 days in total. The materials collected (leaves, roots, and stems) were used for short-term analysis shown in Figures 3–6. Supplementary Figure Legends. Figure S1: methodologies for root phenotyping in tomato. (a) Agar plate, (b) pouch, and (c) rhizotron. Figure S2: comparison of root phenotyping methods in tomato. Root system architecture traits were analyzed in three tomato cultivars under salt or control conditions in agar plates, pouches, or rhizotron: (a) NLR: number of lateral roots; (b) LRL: lateral roots length; (c) Apical: apical zone length; (d) Basal: basal zone length. Plants were growth for 4 days and treated or not with 120 mM NaCl once, for 6 additional days. Roots, of 10-day-old plants, were analyzed with EZ-Rhizo software. Data represent the mean ± SE of 20 replicates from two independent experiments. Figure S3: moderate correlation between salt tolerance parameters and RSA traits. Relationship between ion content parameters in roots treated with salt and RSA traits. (a, b) Na^+^ content. (c–f) K^+^/Na^+^ ratio. RSA traits were represented as the ratio between the salt divided by the control values. Different symbols represent the five genotypes. Correlation between pairs of variables was tested using the Pearson correlation coefficient squared (*R*^2^). Moderate correlations were considered if the value lies between 0.25 and 0.49. Figure S4: reduction of growth parameters by salinity in long-term treatment experiments. (a) Percentage of shoot growth reduction in saline conditions compared to the control. (b) Percentage of flower number reduction per plant in salinity conditions compared to the control. Values represent the mean of several replicates shown as a number above the bars.
