# Supplementary material for: Phenotyping Tomato Root Developmental Plasticity in Response to Salinity in Soil Rhizotrons
Source: Plant Phenomics. 2021 Jan 20;2021:2760532. doi: 10.34133/2021/2760532 (PMC7869940; doi:10.34133/2021/2760532)
Supplement: Supplementary Materials — and Methods S1. For rhizotron phenotyping, two different protocols were carried out. In the preliminary setup (result from Figure 2 and S2), 4-day-old seedlings were transferred to soil plates and irrigated once with 100 ml of water (control) or 100 ml of 120 mM NaCl (salt) solution. RSA was analyzed for roots of 10-day-old plants that were treated for 6 days. In the final setup designed for rhizotron phenotyping and represented graphically in Figure 1(a), 3-day-old seedlings were transferred to soil plates, and 3 days after the transfer to soil, seedlings were irrigated with 100 ml of tap water (control) or 100 ml of 120 mM NaCl (salt) solution per plate. The treatment was repeated every 4 days from the first treatment in the same conditions. In this protocol, two treatments were done, at 6 days and 10 days after germination. Finally, the plants were harvested and analyzed at 14 days old stage and treated for 8 days in total. The materials collected (leaves, roots, and stems) were used for short-term analysis shown in Figures 3–6. Supplementary Figure Legends. Figure S1: methodologies for root phenotyping in tomato. (a) Agar plate, (b) pouch, and (c) rhizotron. Figure S2: comparison of root phenotyping methods in tomato. Root system architecture traits were analyzed in three tomato cultivars under salt or control conditions in agar plates, pouches, or rhizotron: (a) NLR: number of lateral roots; (b) LRL: lateral roots length; (c) Apical: apical zone length; (d) Basal: basal zone length. Plants were growth for 4 days and treated or not with 120 mM NaCl once, for 6 additional days. Roots, of 10-day-old plants, were analyzed with EZ-Rhizo software. Data represent the mean ± SE of 20 replicates from two independent experiments. Figure S3: moderate correlation between salt tolerance parameters and RSA traits. Relationship between ion content parameters in roots treated with salt and RSA traits. (a, b) Na+ content. (c–f) K+/Na+ ratio. RSA traits w [file 2760532.f1.zip › Supplementary_methodsandlegends.docx]

**Supplementary Material and methods S1**

For rhizotrons phenotyping two different protocols were carried out. In the preliminary set up (Result from Figure 1 and S2), 4 days old seedling were transfer to soil plates and irrigated once with 100ml of water (control) or 100ml of 120mM NaCl (Salt) solution. RSA were analysed for roots of 10 days old plants that were treated for 6 days. In the final set up designed for rhizotron phenotyping and represented graphically in the Figure 2a, 3 days old seedlings were transferred to soil plates and 3 days after the transfer to soil, seedlings were irrigated with 100ml of tap water (control) or 100ml of 120mM NaCl (Salt) solution per plate. The treatment was repeated every 4 days from the first treatment in the same conditions. In this protocol two treatment were done, at 6 days and 10 days after germination. Finally, the plants were harvested and analysed at 14 days old stage and treated for 8 days in total. The material collected (leaves, roots and stems) were used for short-term analysis shown in Figure 3-6.

**Supplementary Figure Legends**

**Figure S1**. **Methodologies for root phenotyping in tomato**. **(a)** Agar plate, **(b)** Pouch and **(c)** Rhizotron.

**Figure S2. Comparison of root phenotyping methods in tomato**. Root system architecture traits were analysed in three tomato cultivars under salt or control conditions in agar plates, pouches or rhizotron: **(a)** NLR, number of lateral roots; **(b)** LRL, lateral roots length; **(c)** Apical, apical zone length; **(d)** Basal, basal zone length. Plants were growth for 4 days and treated or not with 120 mM NaCl once, for 6 additional days. Roots, of 10 days old plants were analyzed with EZ-rhizo software. Data represent the mean ± SE of 20 replicates from two independent experiments.

**Figure S3. Moderate correlation between salt tolerance parameters and RSA traits**. Relationship between ion content parameters in roots treated with salt and RSA traits. **(a,b)**  Na^+^ content. **(c-f)** K^+^/Na^+^ ratio. RSA traits were represented as the ratio between the salt divided by the control values. Different symbols represent the five genotype. Correlation between pairs of variables were tested using the Pearson correlation coefficient squared (R^2^). Moderate correlations were considered if the value lies between 0,25 – 0,49.

**Figure S4.** **Reduction of growth parameters by salinity in long-term treatment experiments. (a)** Percentage of shoot growth reduction in saline conditions compared to the control. **(b)** Percentage of flower number reduction per plant in salinity conditions compared to the control. Values represent the mean of several replicates shown as a number above the bars.
