# Supplementary figures and images for: Phenotyping Tomato Root Developmental Plasticity in Response to Salinity in Soil Rhizotrons
Source: Plant Phenomics. 2021 Jan 20;2021:2760532. doi: 10.34133/2021/2760532 (PMC7869940; doi:10.34133/2021/2760532)

## Slide 1
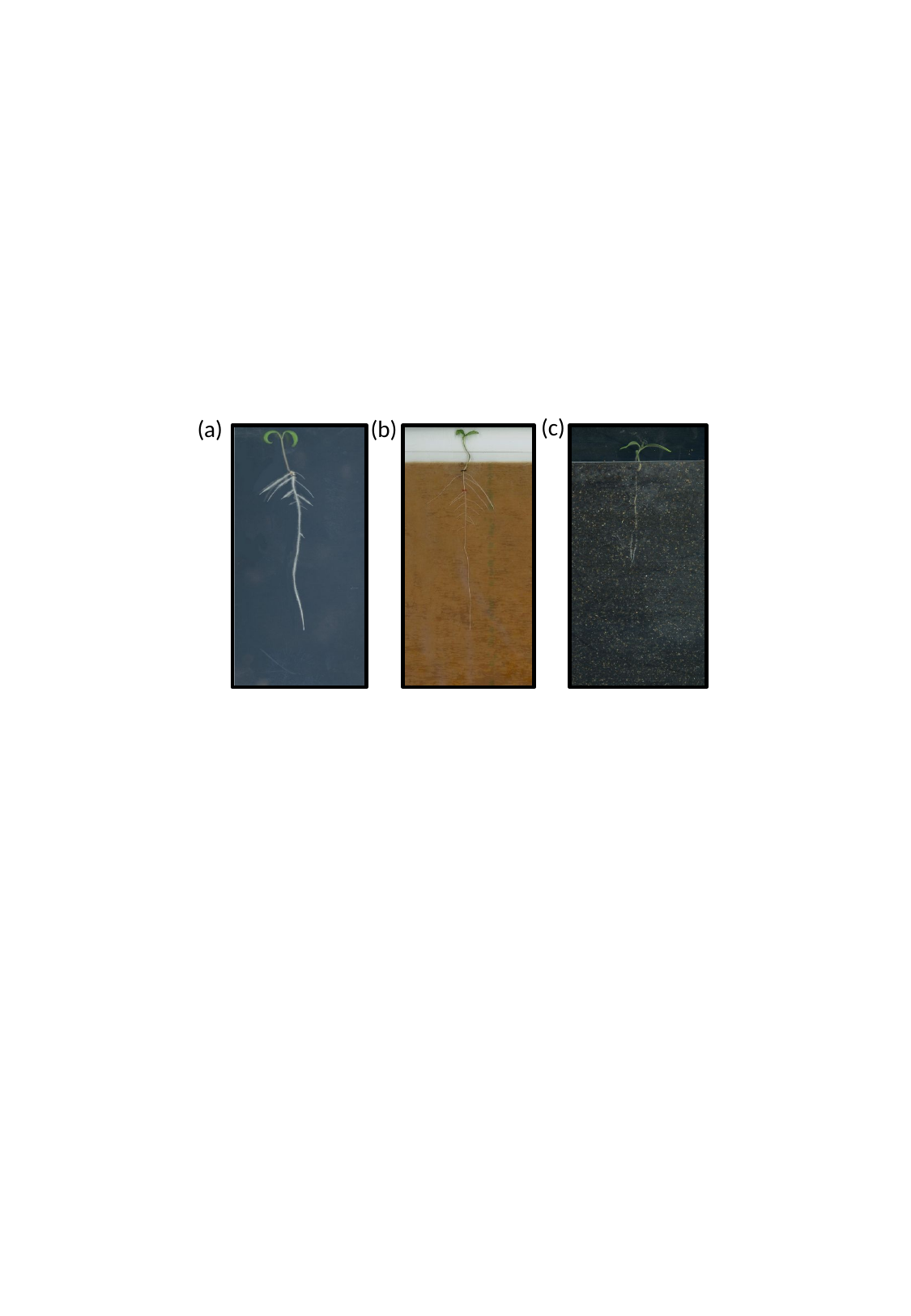

(c)
(a)
(b)

## Slide 2
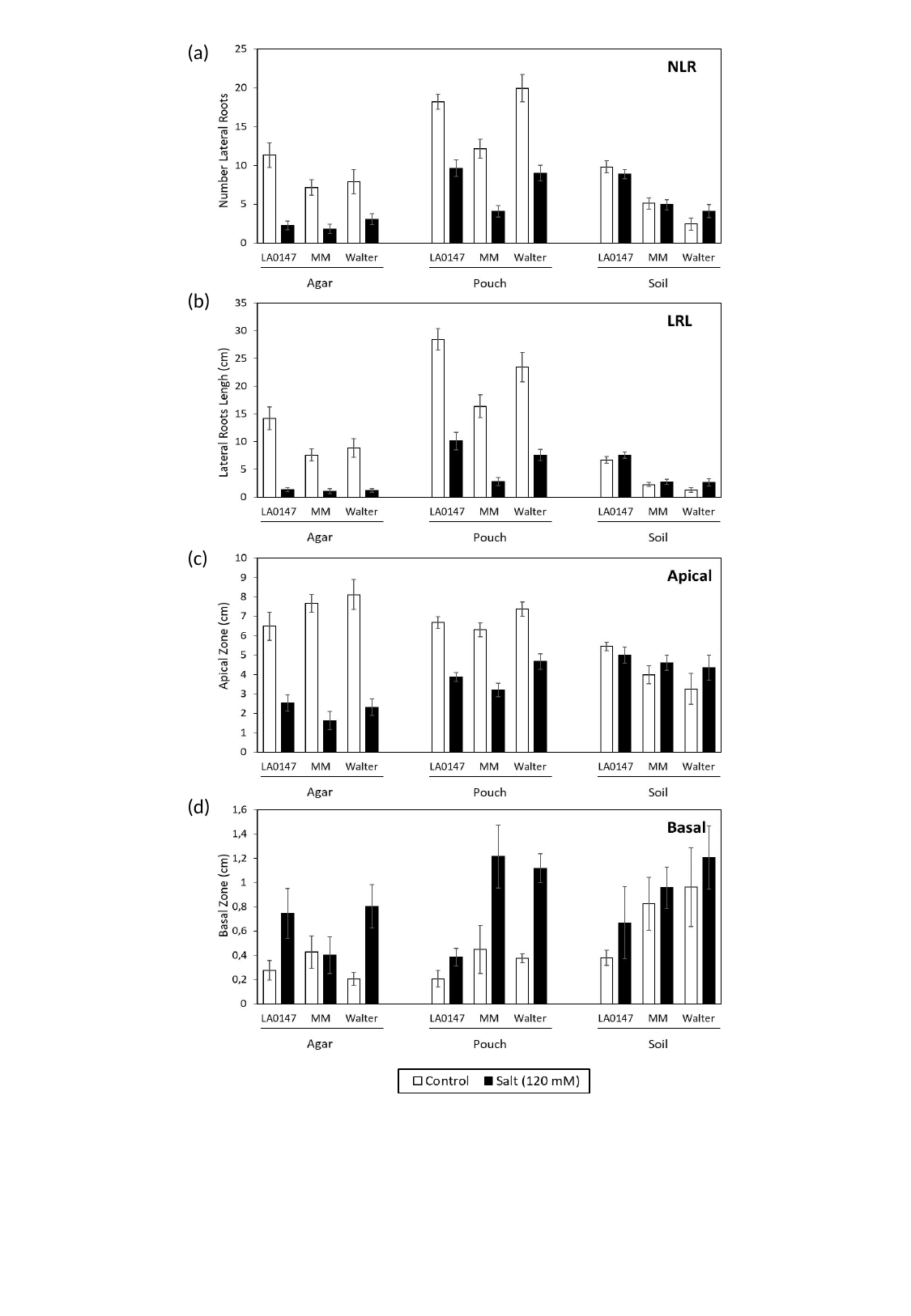

(a)
(b)
(c)
(d)

## Slide 3
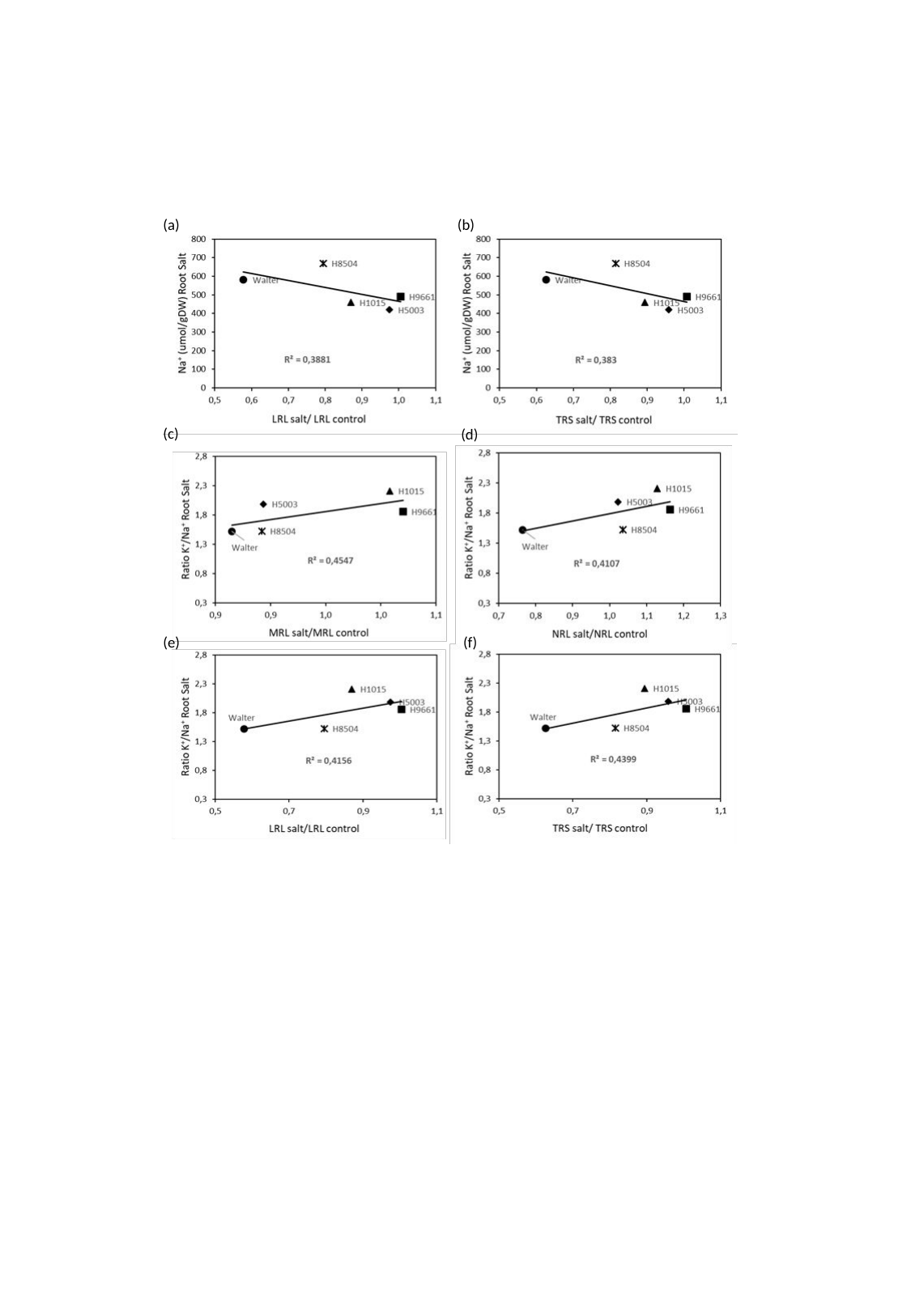

(b)
(a)
(c)
(d)
(f)
(e)

## Slide 4
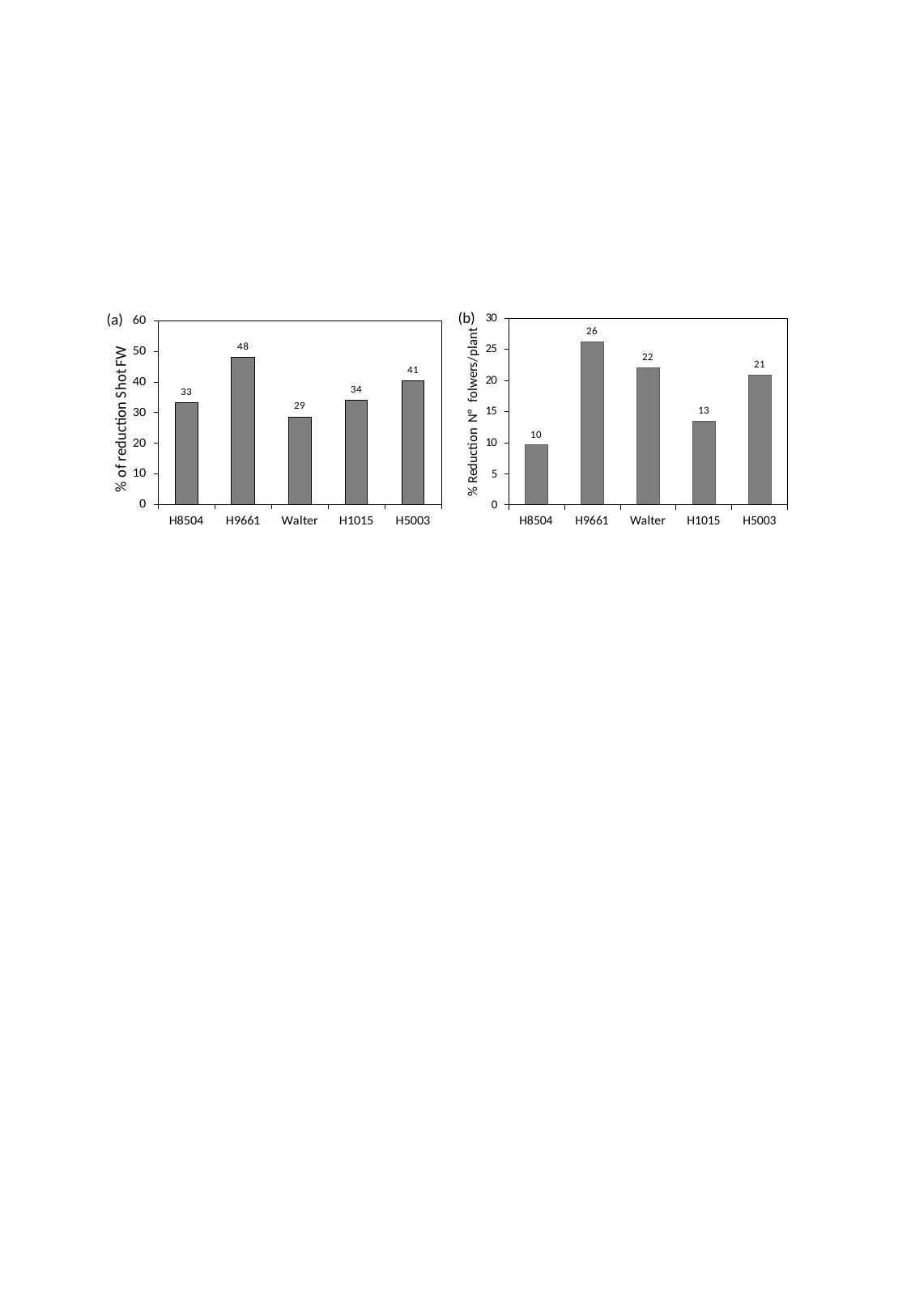

(b)
(a)

Supplement: Supplementary Materials — and Methods S1. For rhizotron phenotyping, two different protocols were carried out. In the preliminary setup (result from Figure 2 and S2), 4-day-old seedlings were transferred to soil plates and irrigated once with 100 ml of water (control) or 100 ml of 120 mM NaCl (salt) solution. RSA was analyzed for roots of 10-day-old plants that were treated for 6 days. In the final setup designed for rhizotron phenotyping and represented graphically in Figure 1(a), 3-day-old seedlings were transferred to soil plates, and 3 days after the transfer to soil, seedlings were irrigated with 100 ml of tap water (control) or 100 ml of 120 mM NaCl (salt) solution per plate. The treatment was repeated every 4 days from the first treatment in the same conditions. In this protocol, two treatments were done, at 6 days and 10 days after germination. Finally, the plants were harvested and analyzed at 14 days old stage and treated for 8 days in total. The materials collected (leaves, roots, and stems) were used for short-term analysis shown in Figures 3–6. Supplementary Figure Legends. Figure S1: methodologies for root phenotyping in tomato. (a) Agar plate, (b) pouch, and (c) rhizotron. Figure S2: comparison of root phenotyping methods in tomato. Root system architecture traits were analyzed in three tomato cultivars under salt or control conditions in agar plates, pouches, or rhizotron: (a) NLR: number of lateral roots; (b) LRL: lateral roots length; (c) Apical: apical zone length; (d) Basal: basal zone length. Plants were growth for 4 days and treated or not with 120 mM NaCl once, for 6 additional days. Roots, of 10-day-old plants, were analyzed with EZ-Rhizo software. Data represent the mean ± SE of 20 replicates from two independent experiments. Figure S3: moderate correlation between salt tolerance parameters and RSA traits. Relationship between ion content parameters in roots treated with salt and RSA traits. (a, b) Na+ content. (c–f) K+/Na+ ratio. RSA traits w [file 2760532.f1.zip › Suplemental Figures_all.pptx]
